# Supplementary material for: Molecular mechanism for Rabex-5 GEF activation by Rabaptin-5
Source: eLife. 2014 Jun 23;3:e02687. doi: 10.7554/eLife.02687 (PMC4102244; doi:10.7554/eLife.02687)
Supplement: Figure 1—source data 1. — DOI: http://dx.doi.org/10.7554/eLife.02687.005 [file elife02687s001.doc]

**Figure 1-Source data 1. Interactions between Rabex-5CC and Rabaptin-5C21 in the Rabex-5Δ-Rabaptin-5C21_2_ complex.**

**(A) Hydrogen bonds and salt bridges (≤ 3.5 Å)**

**Rabex-5CC Distance (Å) Rabaptin-5C21^A^ Rabaptin-5C21^B^**

Arg423-N^η1^ 2.7 Glu607-O^ε1^

Gln424-N^ε2^ 3.2 Leu599-O

Glu425-O^ε1^ 3.2 Arg602-N^η1^

Glu425-O^ε1^ 2.8 Arg602-N^η2^

Gln450-N^ε2^ 3.3 Gln627-O^ε1^

**(B) van der Waals contacts (≤ 4.0 Å)**

**Rabex-5CC Rabaptin-5C21^A^ Rabaptin-5C21^B^**

Leu420 (5) ^a^ Ile596 (2) Leu599 (2)

Val600 (1)

Asn421 (3) Leu599 (3)

Arg423 (4) Glu607 (4)

Gln424 (12) Leu599 (4)

Arg602 (5)

Ala603 (3)

Glu425 (3) Arg602 (3)

Ile427 (10) Ser606 (2) Ser606 (3)

Glu607 (3)

Leu610 (2)

Met428 (6) Arg602 (4)

Ser606 (1)

Leu609 (1)

Glu430 (4) Leu610 (2)

Gln614 (2)

Ala431 (4) Leu610 (1) Leu609 (2)

Leu613 (1)

Lys432 (3) Leu609 (3)

Leu434 (5) Leu613 (3)

Gln614 (1)

Leu617 (1)

Glu435 (3) Leu613 (3)

Asp437 (1) Leu617 (1)

Leu438 (6) Leu617 (2) Gly616 (3)

Leu617 (1)

Trp441 (20) Leu617 (1) Ala620 (5)

Ala620 (3) Val624 (6)

Lys621 (3)

Val624 (2)

Thr442 (2) Ala620 (2)

Ile445 (6) Val624 (1) Val624 (3)

Met628 (2)

Ala446 (7) Gln627 (7)

Glu448 (1) Met628 (1)

Val449 (6) Gln627 (3)

Val630 (1)

Leu631 (2)

Gln450 (4) Gln627 (4)

Ile452 (1) Leu631 (1)

^a^ There are a total of 116 van der Waals contacts. Number in parentheses refers to the number of van der Waals contacts the residue is involved.
