# Supplementary material for: Molecular mechanism for Rabex-5 GEF activation by Rabaptin-5
Source: eLife. 2014 Jun 23;3:e02687. doi: 10.7554/eLife.02687 (PMC4102244; doi:10.7554/eLife.02687)
Supplement: Figure 3—source data 1. — DOI: http://dx.doi.org/10.7554/eLife.02687.016 [file elife02687s003.doc]

**Figure 3-Source data 1. SAXS analysis parameter**s

| **Maximum paired-distance (D_max_, Å)** | | | | |
| --- | --- | --- | --- | --- |
| **Protein** | **Experimental** | **Calculated** | | |
|  |  | **Model** | **Dimer** | **Monomer** |
| R2 | 179.1 | R2Δ V-shaped | 151.5 | 110.5 |
| R2Δ | 169.2 | R2Δ linear | 150.5 | 150.5 |
| R3 | 159.2 | R3Δ | 150.5 | 150.5 |
| R3Δ | 179.1 |  |  | |
| **Radius of gyration (Rg, Å)** | | | | |
| **Protein** | **Experimental** | **Calculated** | | |
|  |  | **Model** | **Dimer** | **Monomer** |
| R2 | 45.2 ± 3.9 | R2Δ V-shaped | 45.0 | 29.4 |
| R2Δ | 45.3 ± 4.3 | R2Δ linear | 42.4 | 38.1 |
| R3 | 45.4 ± 2.7 | R3Δ | 41.4 | 35.0 |
| R3Δ | 41.1 ± 1.8 |  |  | |
|  | | | | |
| **Protein** | **Porod volume (nm^3^)** | **Protein** | **Molecular mass (kDa)** | |
|  |  |  | **Dimer** | **Monomer** |
| R2 | 210 | R2 | 118 | 59 |
| R2Δ | 200 | R2Δ | 114 | 57 |
| R3 | 295 | R3 | 156 | 78 |
| R3Δ | 295 | R3Δ | 152 | 76 |
