# Supplementary material for: Molecular mechanism for Rabex-5 GEF activation by Rabaptin-5
Source: eLife. 2014 Jun 23;3:e02687. doi: 10.7554/eLife.02687 (PMC4102244; doi:10.7554/eLife.02687)
Supplement: Figure 4—source data 1. — DOI: http://dx.doi.org/10.7554/eLife.02687.019 [file elife02687s004.doc]

**Figure 4-Source data 1. GEF activity of different Rabex-5 variants alone and in complexes with different Rabaptin-5C21 mutants or truncates.**

|  | **Catalytic efficiency (×10^4^ M^-1^·s^-1^)** |
| --- | --- |
| Rabex-5 GEF | 2.93 ± 0.06 |
| Rabex-5 | 0.93 ± 0.03 |
| Rabex-5Δ | 1.76 ± 0.07 |
| R2 ^a^ | 3.07 ± 0.08 |
| R2Δ | 2.88 ± 0.07 |
| R2M1 | 2.98 ± 0.09 |
| R2M2 | 2.78 ± 0.11 |
| R2ΔN | 1.02 ± 0.05 |

^a^ The R2 complex was co-expressed and co-purified.

R2M1: the R2 complex in which Rabaptin-5C21 contains a quadruple mutation N568A/E572A/Q579A/E582A;

R2M2: the R2 complex in which Rabaptin-5C21 contains a double mutation I608A/D623A;

R2ΔN: the R2 complex in which the N-terminal residues 552-592 of Rabaptin-5C21 were deleted.
