# Supplementary material for: Molecular mechanism for Rabex-5 GEF activation by Rabaptin-5
Source: eLife. 2014 Jun 23;3:e02687. doi: 10.7554/eLife.02687 (PMC4102244; doi:10.7554/eLife.02687)
Supplement: Figure 4—source data 2. — DOI: http://dx.doi.org/10.7554/eLife.02687.020 [file elife02687s005.doc]

**Figure 4-Source data 2. GEF activity of different Rabex-5 mutants alone and in complexes with wild-type Rabaptin-5C21.**

|  | **Catalytic efficiency (×10^4^ M^-1^·s^-1^)** |
| --- | --- |
| Rabex-5 GEF | 2.93 ± 0.06 |
| Rabex-5 | 0.93 ± 0.03 |
| R2 WT ^a^ | 1.46 ± 0.05 |
| Rabex-5 N413A | 1.81 ± 0.10 |
| Rabex-5 L414A | 1.65 ± 0.06 |
| Rabex-5 L417A | 1.29 ± 0.06 |
| Rabex-5 L420A | 1.73 ± 0.06 |
| Rabex-5 N421A | 1.05 ± 0.05 |
| Rabex-5 R423A | 1.72 ± 0.07 |
| Rabex-5 I427A | 1.71 ± 0.04 |
| Rabex-5 L434D | 0.83 ± 0.04 |
| R2 L434D | 0.87 ± 0.03 |
| Rabex-5 L438D | 0.88 ± 0.03 |
| R2 L438D | 1.01 ± 0.03 |
| Rabex-5 I439D | 0.98 ± 0.05 |
| R2 I439D | 1.47 ± 0.05 |
| Rabex-5 W441A | 1.02 ± 0.04 |
| R2 W441A | 1.06 ± 0.03 |

^a^ The R2 complex was prepared by mixing the individually purified Rabex-5 and Rabaptin-5C21 together.
