# Supplementary material for: Molecular mechanism for Rabex-5 GEF activation by Rabaptin-5
Source: eLife. 2014 Jun 23;3:e02687. doi: 10.7554/eLife.02687 (PMC4102244; doi:10.7554/eLife.02687)
Supplement: Figure 4—source data 3. — DOI: http://dx.doi.org/10.7554/eLife.02687.021 [file elife02687s006.doc]

**Figure 4-Source data 3. GEF activity of wild-type Rabex-5 in complexes with different Rabaptin-5C21 mutants.**

|  | **Catalytic efficiency (×104 M^-1^·s^-1^)** |
| --- | --- |
| Rabex-5 | 0.93 ± 0.03 |
| R2 WT ^a^ | 1.46 ± 0.05 |
| R2 I608D | 1.52 ± 0.08 |
| R2 L610D | 0.99 ± 0.03 |
| R2 L613D | 0.95 ± 0.03 |
| R2 L617D | 0.99 ± 0.03 |

^a^ The R2 complex was prepared by mixing the individually purified Rabex-5 and Rabaptin-5C21 together.
